# Effects of a Torsion-Adaptive Energy-Storing-and-Return Prosthetic Foot on Balance Control in Individuals with Transtibial Amputation: A Pilot Pre–Post Motion Analysis Study

**DOI:** 10.3390/bioengineering13080949

**Published:** 2026-08-21

**Authors:** Ho-Yong Jeong, Hee Seung Yang, Yea-Eun Lee, Pyoung-hwa Choi, Chan-hyeok Jeong, Hui-Woo Choi, Young Lee

**Affiliations:** 1 Department of Physical Medicine and Rehabilitation, Veterans Health Service Medical Center, Seoul 05368, Republic of Korea; ghdyd8623@gmail.com (H.-Y.J.); kongmom0819@hanmail.net (Y.-E.L.); 2 Veterans Health Service Medical Center, Center of Prosthetics and Orthotics, Seoul 05368, Republic of Korea; peace8422@naver.com; 3 Department of Physical Education, Graduate School, Korea National Sport University, Seoul 05541, Republic of Korea; chj3288@gmail.com; 4 Department of Rehabilitation Medical Technology, Korea Nazarene University, Cheonan 31172, Republic of Korea; hwhw2130@gmail.com; 5 Veterans Medical Research Institute, Veterans Health Service Medical Center, Seoul 05368, Republic of Korea; lyou7688@gmail.com

**Keywords:** transtibial amputation, energy-storing-and-return foot, postural stability, balance control, 3D motion analysis

## Abstract

Individuals with transtibial amputation have impaired postural control due to limited ankle function. Conventional energy-storing-and-return (ESAR) prosthetic feet mainly support sagittal-plane mechanics and provide limited frontal-plane adaptability. This pilot pre–post motion analysis study examined the effects of the Intersection Foot^®^ (IF), a torsion-adaptive ESAR prosthetic foot, on balance control in 10 male individuals with unilateral transtibial amputation. Three-dimensional motion analysis and force plate measurements were performed during five Berg Balance Scale-derived tasks. During sit-to-stand, IF reduced the mediolateral root mean square distance of center of pressure from 10.20 ± 3.59 to 8.29 ± 3.32 mm (*p* = 0.047) and the anteroposterior center of mass (CoM) range from 345.51 ± 31.72 to 326.45 ± 47.40 mm (*p* = 0.008). During tandem stance, IF increased mediolateral CoM range from 23.94 ± 18.77 to 55.99 ± 30.90 mm (*p* = 0.021), vertical CoM range from 4.14 ± 4.18 to 9.45 ± 7.72 mm (*p* = 0.038), and sound-side frontal-plane ankle ROM from 1.44 ± 1.20° to 2.48 ± 1.30° (*p* = 0.022). Overall, replacing the habitual feet with IF produced task-dependent biomechanical adaptations rather than uniform improvements in postural stability, suggesting reduced dynamic sway during sit-to-stand but increased whole-body and sound-limb compensatory motion during tandem stance.

## 1. Introduction

Individuals with lower-limb amputation experience impaired postural control partly because limb loss disrupts somatosensory feedback and alters lower-limb mechanics [1,2]. In particular, the absence of active ankle joint function necessitates greater reliance on proximal compensatory strategies involving the hip, pelvis and trunk to maintain stability [3,4]. These compensatory strategies are characterized by asymmetric weight bearing, with greater reliance on the intact limb during standing and balance tasks [2,5]. In addition, individuals with lower-limb amputation may develop secondary musculoskeletal complications, including hip or knee flexion contractures, which can complicate prosthetic fitting and contribute to altered walking biomechanics and gait deviations [6]. These postural control deficits are clinically important because falls remain common among lower-limb prosthesis users, with approximately 48–60% reporting one or more falls annually [7,8]. Moreover, lower balance confidence, poorer functional mobility, and reduced prosthetic-side reach have been associated with recurrent falls in adults with lower-limb amputation [9]. Accordingly, prosthetic ankle–foot designs and mechanical properties that better support postural control may contribute to improved functional stability in individuals with lower-limb amputation [10,11,12].

Mediolateral stability is particularly challenging in individuals with transtibial amputation because the prosthetic side lacks active ankle-mediated modulation of the center of pressure (CoP) [13]. During outward-directed perturbations delivered at prosthetic-side foot contact, individuals with transtibial amputation showed no in-stance corrective response in CoP or ground reaction force (GRF) [13]. Instead, they relied on a cross-step with the sound limb, resulting in greater center of mass (CoM) displacement. This limitation in distal frontal-plane control increases reliance on proximal and stepping strategies. During step ascent and descent, individuals with transtibial amputation have demonstrated greater lateral trunk flexion and altered hip-abductor loading patterns [14]. Consistent with the clinical importance of these proximal strategies, recent evidence showed that residual-limb hip-abductor torque steadiness explained approximately half of the variance in multidirectional and mediolateral balance performance [15]. Collectively, these findings highlight frontal-plane balance recovery as a clinically relevant target and provide a rationale for investigating prosthetic designs that offer greater mediolateral adaptability.

Conventional passive energy-storing-and-return (ESAR) prosthetic feet store and return energy through the elastic deformation of their structural components. Traditionally, their design and mechanical evaluation have emphasized sagittal-plane functions, such as plantarflexion–dorsiflexion mechanics, roll-over characteristics, and forward progression [16,17]. To improve mediolateral accommodation, several frontal-plane strategies have been investigated. These include modifications of frontal-plane stiffness [18], split-toe configurations and articulated or adaptive frontal-plane mechanisms [19,20], and powered two-degree-of-freedom (2-DoF) ankle–foot systems capable of active inversion–eversion [21]. These approaches rely on different mechanical principles: stiffness-based designs modify resistance to mediolateral deformation and articulated or polycentric mechanisms permit passive frontal-plane motion, whereas powered systems actively generate inversion–eversion torque [18,19,20,21]. Mechanical and human-subject studies indicate that these approaches can alter frontal-plane motion, lateral forces, inversion–eversion moments, and mediolateral balance; however, their effects on proximal compensatory control and whole-body stability are not uniform [18,20,22]. In a recent motion analysis study, a frontal-plane adaptable linkage increased prosthetic ankle frontal-plane motion during level walking, unstable-surface walking, and side-stepping, but it did not produce corresponding improvements in step width, walking velocity, or CoM deviation [22]. Collectively, these findings indicate that increased frontal-plane prosthetic mobility does not necessarily translate into improved proximal control or whole-body stability.

**Figure 1:**
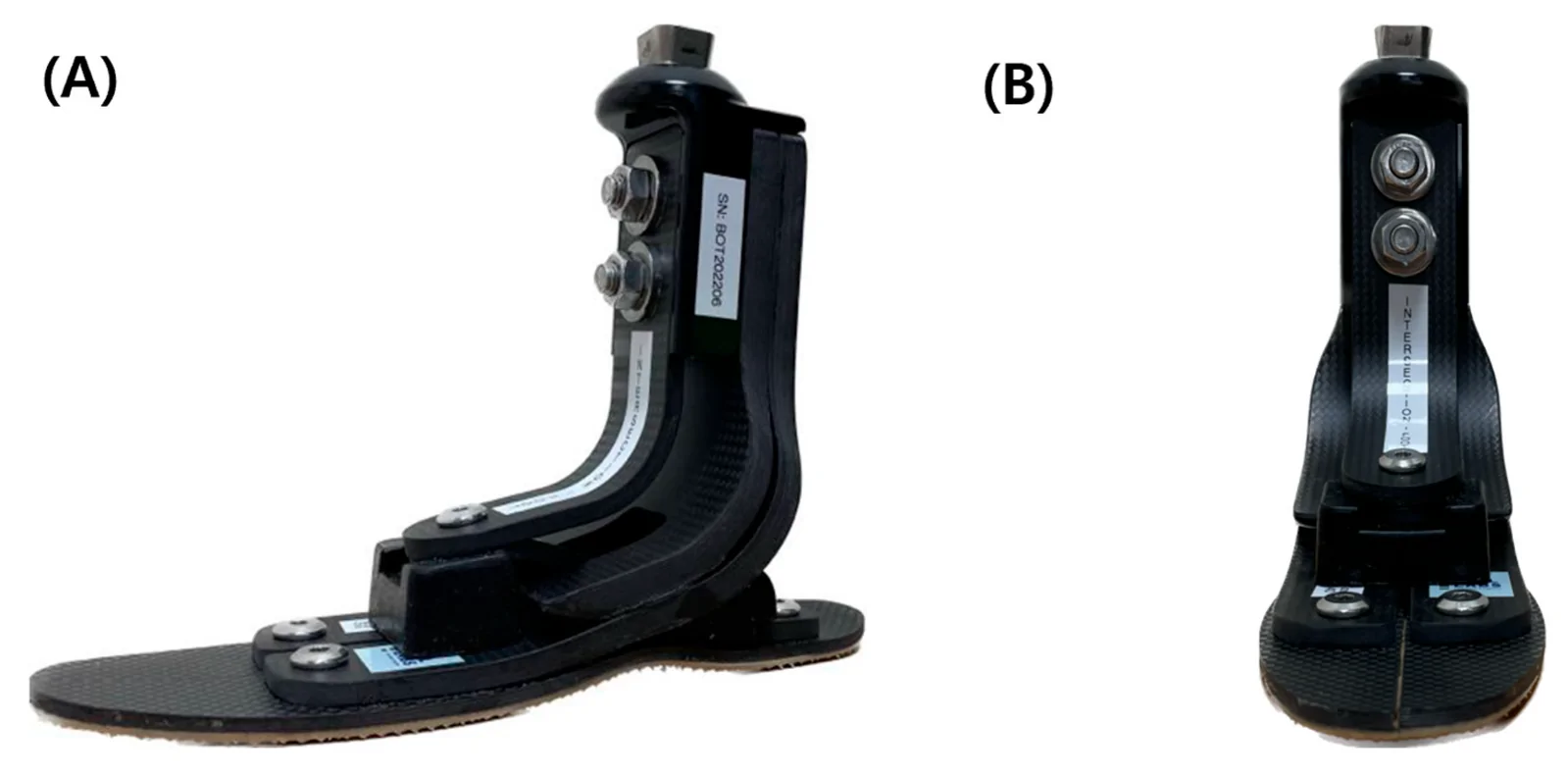
Structural design of the Intersection Foot^®^ (IF). (**A**) Lateral view and (**B**) anterior view of the prosthetic foot, showing its asymmetric medial–lateral carbon-fiber structure.

Against this background, the Intersection Foot^®^ (IF; Boilabs Inc., Korea) was developed as a passive torsion-adaptive ESAR prosthetic foot (Figure 1). Rather than using a powered actuator or a discrete articulated ankle mechanism to generate frontal-plane motion, the IF is designed to obtain mediolateral and torsional adaptability through its asymmetric carbon-fiber geometry and load-dependent elastic deformation. As an ESAR structure, the carbon-fiber components are intended to store elastic energy during loading and return this energy during unloading. Specifically, the IF incorporates an asymmetric medial–lateral configuration, consisting of three carbon-fiber columns on the medial side and two columns on the lateral side. This configuration is intended to permit greater elastic deformation medially during weight bearing while providing relatively greater structural support laterally. In addition, its X-shaped plantar configuration is designed to allow load-dependent mediolateral and torsional deformation without a separate inversion–eversion joint. Although the specific mechanical behavior of the IF has not yet been independently quantified in peer-reviewed studies, previous investigations of passive prosthetic feet have shown that structural features such as split-toe geometry and frontal-plane adaptable mechanisms can modify lateral displacement, mediolateral forces, and inversion–eversion moments during cross-slope loading [19,20,23]. Therefore, the IF represents a structurally adaptive approach in which frontal-plane and torsional compliance are incorporated within the ESAR foot architecture itself.

However, the effects of such structural adaptability on postural control during clinically relevant static and transitional balance tasks remain unclear. The Berg Balance Scale (BBS) is an established clinical measure of functional balance in individuals with lower-limb amputation [24]; however, its ordinal scoring does not directly quantify task-specific biomechanical responses. Therefore, the present study quantified selected BBS-derived balance tasks using three-dimensional motion analysis and force plate measurements. The primary aim was to determine whether replacing participants’ habitual prosthetic feet with the IF was associated with task-specific changes in postural control. We hypothesized that the torsion-adaptive structural configuration of the IF would alter mediolateral postural control responses, particularly during tasks requiring greater frontal-plane control.

## 2. Materials and Methods

### 2.1. Design and Setting

This pilot study used a single-group, within-subject pre–post design and was conducted at the Department of Rehabilitation Medicine and the Prosthetics and Orthotics Center of the Veterans Health Service Medical Center, Seoul, Republic of Korea. Participants were recruited between April and November 2022. The study protocol was approved by the Institutional Review Board of the Veterans Health Service Medical Center (IRB File No. 2022-03-035) and was conducted in accordance with the Declaration of Helsinki. Written informed consent was obtained from all participants before enrollment.

### 2.2. Participants

Eligibility criteria were predefined in the study protocol to ensure safe participation in weight-bearing balance assessments and to minimize conditions that could substantially affect gait or balance. The inclusion criteria were as follows: (1) age ≥ 19 years; (2) unilateral transtibial amputation; (3) at least 6 months since amputation; (4) continuous use of the current prosthesis for at least 3 months before enrollment; and (5) a functional ambulation level of K2 or higher. Functional ambulation level was assessed by a board-certified physiatrist using the Medicare Functional Classification Level (K-level) criteria. Participants were required to have a K-level of 2 or higher, indicating the ability to ambulate independently and negotiate low-level environmental barriers, such as curbs or uneven surfaces.

The exclusion criteria were as follows: (1) cognitive impairment precluding independent decision-making or participation in the assessments; (2) severe lower-limb joint contracture, untreated fracture, or severe osteoporosis contraindicating weight bearing; (3) history of lower-limb orthopedic surgery within the previous 6 months; (4) cardiovascular or respiratory conditions that could be exacerbated by physical exertion; (5) musculoskeletal pain in body regions other than the amputated limb that could substantially affect gait or balance; (6) upper-limb amputation; and (7) any other condition considered by the investigators to make participation inappropriate or unsafe.

### 2.3. Study Procedure

This study used a within-subject pre–post design. At baseline, each participant was evaluated while wearing their habitual conventional prosthetic foot (CPF). Following the baseline assessment, a certified prosthetist replaced the CPF with the IF. To isolate the effect of the prosthetic foot, the existing socket and suspension system were maintained, and only the foot component was replaced. Static and dynamic alignment was adjusted by a certified prosthetist using conventional clinical alignment procedures [25]. Participants then used the IF for a 4-week accommodation period before the post-intervention assessment. This period was provided to allow participants to become accustomed to the mechanical characteristics of the new prosthetic foot and was consistent with accommodation periods used in previous prosthetic foot studies [26,27]. Before each motion analysis session, participants were provided with a 30 min familiarization period with the testing environment and task procedures.

### 2.4. Balance Tasks and Experimental Setup

Balance tasks were selected from BBS, an established clinical assessment of functional balance that has demonstrated validity and reliability in individuals with lower-limb amputation [24,28]. BBS comprises 14 functional tasks involving sitting, standing, transfers, and postural transitions, with each item scored on an ordinal scale from 0 to 4 [28]. For the present biomechanical assessment, five BBS-derived tasks that involved foot contact with the ground and were suitable for three-dimensional motion analysis and force plate measurements were selected: (1) sit-to-stand, representing a transitional movement requiring dynamic postural control; and four static balance conditions: (2) standing with eyes open, (3) standing with eyes closed, (4) standing with feet together, and (5) tandem stance with the sound limb positioned in front. To minimize the potential influence of task-related fatigue, a 3 min rest period was provided between tasks.

**Figure 2:**
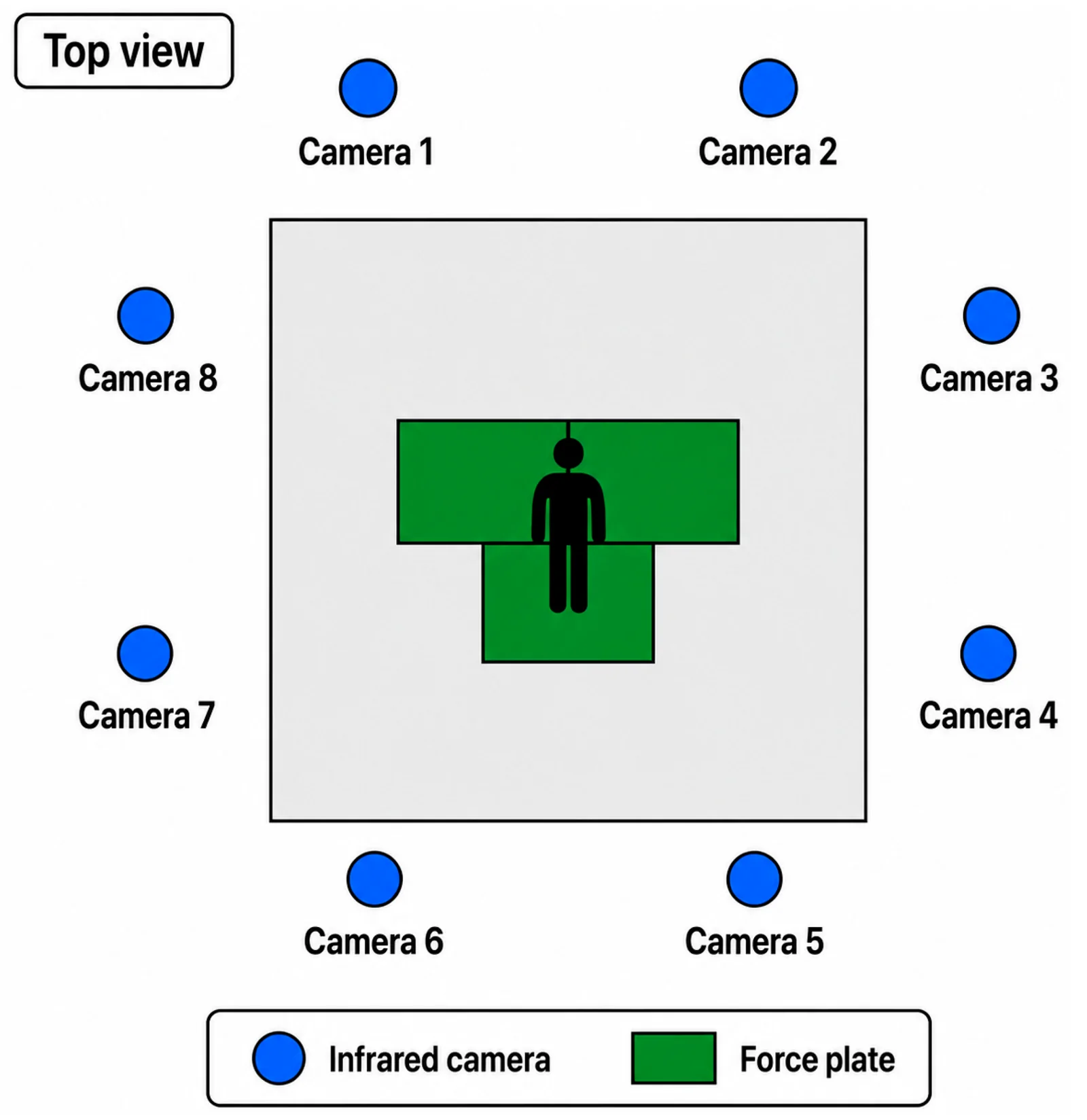
Experimental setup for three-dimensional motion analysis and force plate measurements. The top-view schematic illustrates the arrangement of eight infrared cameras surrounding the testing area and three adjacent force plates positioned at the center in a T-shaped configuration. The participant stood on the force plates during the balance-task assessments.

**Figure 3:**
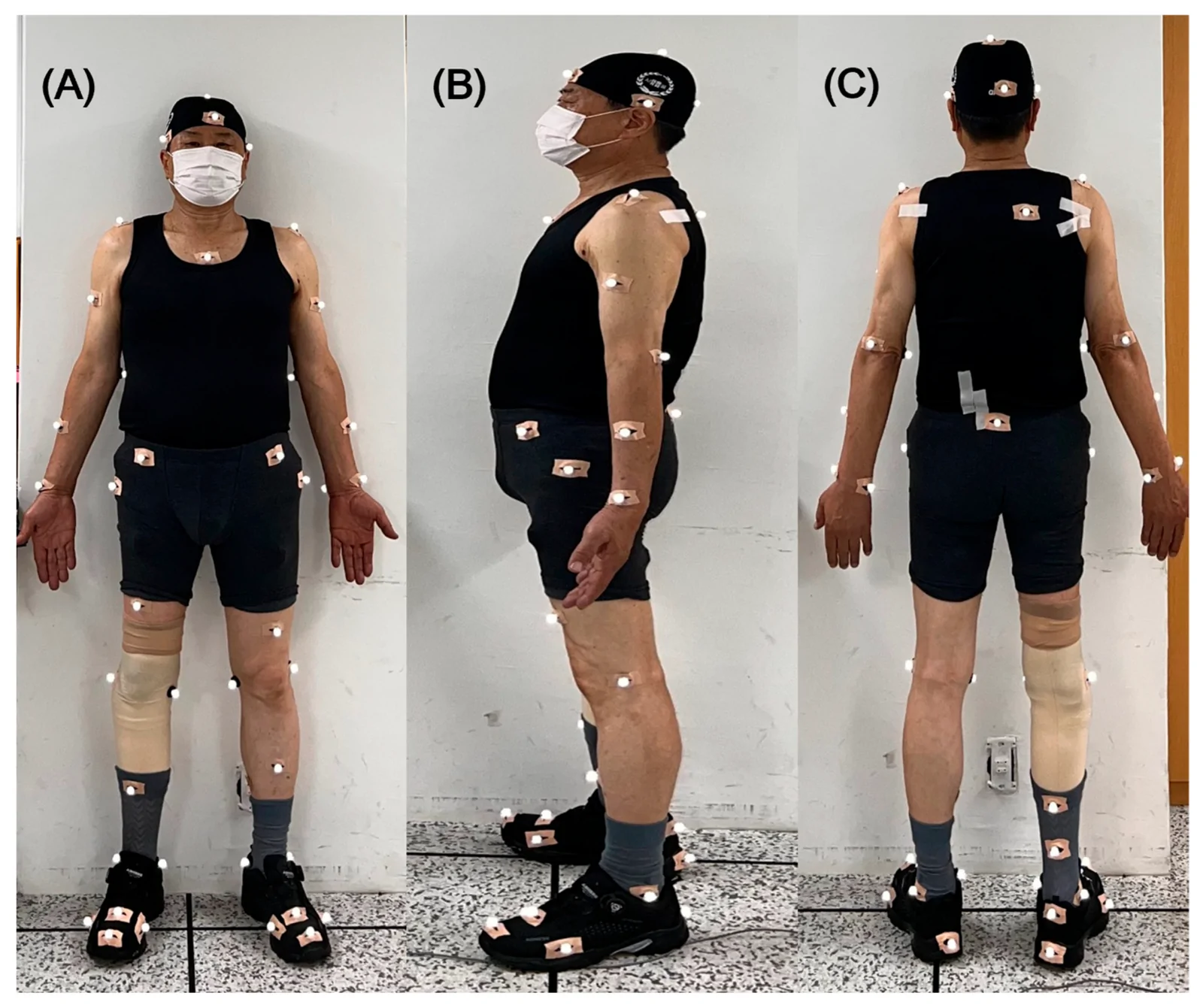
Representative full-body marker placement for three-dimensional motion analysis in an individual with unilateral transtibial amputation. (**A**) Anterior view, (**B**) lateral view, and (**C**) posterior view. Reflective markers were attached to predefined anatomical landmarks and corresponding prosthetic components to estimate segmental kinematics and approximate joint centers.

The selected BBS tasks were adapted for standardized laboratory biomechanical assessment rather than administered for conventional BBS scoring. During the sit-to-stand task, participants folded their arms across their chest and rose from an armless chair at a self-selected comfortable speed. Seat height was individually adjusted to achieve approximately 90° knee flexion [29]. During the static standing tasks, participants maintained their arms comfortably at their sides. For the eyes-open condition, participants fixed their gaze on a stationary point directly ahead; for the eyes-closed condition, participants closed their eyes without a blindfold. During tandem stance, the prosthetic limb was consistently positioned as the rear limb to provide a standardized limb configuration and to permit consistent evaluation of the prosthetic foot condition across participants and between the CPF and IF assessments. The 3D motion analysis was conducted using eight infrared cameras (Raptor-12HS, Motion Analysis Corp., Rohnert Park, CA, USA) at a sampling rate of 100 Hz and three force plates (Kistler Corp., Amherst, NY, USA) at a sampling rate of 1200 Hz (Figure 2). Camera calibration was performed using the non-linear transformation method. All motion capture systems were synchronized via an internal trigger using an analog-to-digital board. Data acquisition and processing were performed using the Cortex software (version 5.5.0; Motion Analysis Corp., Rohnert Park, CA, USA). Reflective markers with a diameter of 12.5 mm were placed on the predefined anatomical landmarks using a modified Helen–Hayes marker set. For the prosthetic limb, markers were placed on the corresponding prosthetic components to approximate anatomical landmark locations. Specifically, ankle markers were placed on the prosthetic side at a height corresponding to the medial and lateral malleoli of the contralateral sound limb [30]. A representative full-body marker placement, including the prosthetic limb, is shown in Figure 3.

### 2.5. Data Processing and Outcome Variables

**Figure 4:**
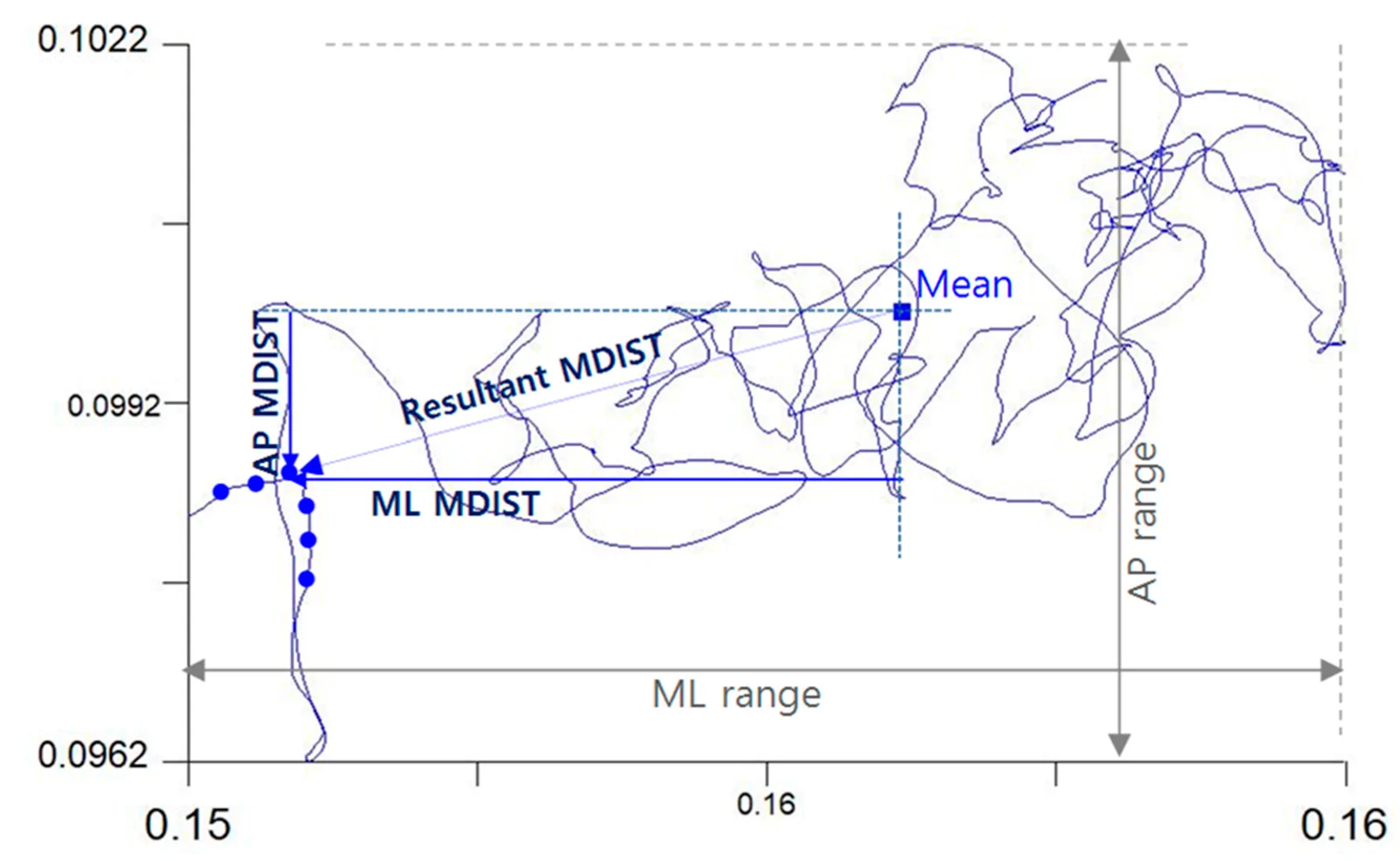
Representative center of pressure (CoP) trajectory and CoP range and MDIST variables. The blue trajectory indicates CoP displacement during a balance task, and the cross indicates the mean CoP position. The mediolateral (ML) and anteroposterior (AP) ranges represent the maximum CoP displacements in the ML and AP directions, respectively. The ML, AP, and resultant mean distances (MDISTs) represent the mean distances from the mean CoP position in each direction and in the AP–ML plane. The dashed guide lines indicate the mean CoP coordinates and the AP and ML extrema used to illustrate the corresponding range measures.

Prior to calculating the outcome measures, kinematic and force plate data were filtered using a low-pass Butterworth filter with a cut-off frequency of 6 Hz. The analyzed variables were computed during the five selected BBS tasks and encompassed spatiotemporal and kinematic measures related to the center of pressure (CoP) and center of mass (CoM). The CoP variables derived from force plate data included the range of CoP displacement in the anteroposterior (AP) and ML directions. Additionally, the mean distance (MDIST) and root mean square distance (RDIST) of the CoP were calculated for both the AP and ML directions, as well as the resultant magnitude, which represented the overall magnitude of postural sway independent of direction (Figure 4).

The CoM variables calculated from the full-body kinematic model included the range of CoM movements in the AP, ML, and vertical directions, providing a macro-level assessment of systemic postural stability.

To evaluate the compensatory strategies of the lower limbs, joint kinematics were analyzed. Specifically, ankle range of motion (ROM) in both the sagittal and frontal planes was compared between the amputated and sound sides. Finally, for the tandem stance task, the balance maintenance time (s) was recorded to quantify the overall task performance.

### 2.6. Statistical Analysis

Given the exploratory pilot nature of this study, no formal a priori power-based sample size calculation was performed. A sample of 10 participants was included to obtain preliminary biomechanical data on the effects of the IF. Owing to the relatively small sample size (*n* = 10) and paired study design, nonparametric statistical methods were used. Differences in biomechanical variables between the CPF and IF conditions were analyzed using a two-sided Wilcoxon signed-rank test based on a normal approximation without continuity correction. To quantify the magnitude and direction of within-subject differences, effect sizes were calculated as Hedges’ *g* based on paired differences, incorporating a correction for small-sample bias, with corresponding 95% confidence intervals (CIs). The magnitude of the effect sizes was interpreted based on the absolute value of Hedges’ *g* as small (|*g*| = 0.2), moderate (|*g*| = 0.5), or large (|*g*| = 0.8). Positive Hedges’ *g* values indicate higher values in the CPF condition, whereas negative values indicate higher values in the IF condition. All statistical analyses were conducted using the R software (version 4.1.2; R Foundation for Statistical Computing, Vienna, Austria). Statistical significance was defined as a two-sided *p*-value of <0.05.

## 3. Results

The demographic and prosthetic characteristics of the 10 male participants are summarized in Table 1. The participants had a mean age of 65.9 years, a mean height of 170.7 cm, a mean body mass of 72.15 kg, a mean residual tibial length of 15.15 cm, and a mean time since amputation of 34.8 years. The causes of amputation were trauma (*n* = 9) and vascular disease (*n* = 1). Functional ambulation levels consisted of K2 (*n* = 2) and K3 (*n* = 8). Regarding baseline prosthetic components, foot types included ESAR (*n* = 9) and single-axis (*n* = 1). Socket types were total surface bearing (TSB, *n* = 9) and patellar tendon bearing (PTB, *n* = 1), and suspension systems utilized suction (*n* = 5), pin lock (*n* = 4), and supracondylar cuff (*n* = 1).

### 3.1. Sit-to-Stand Task

**Figure 5:**
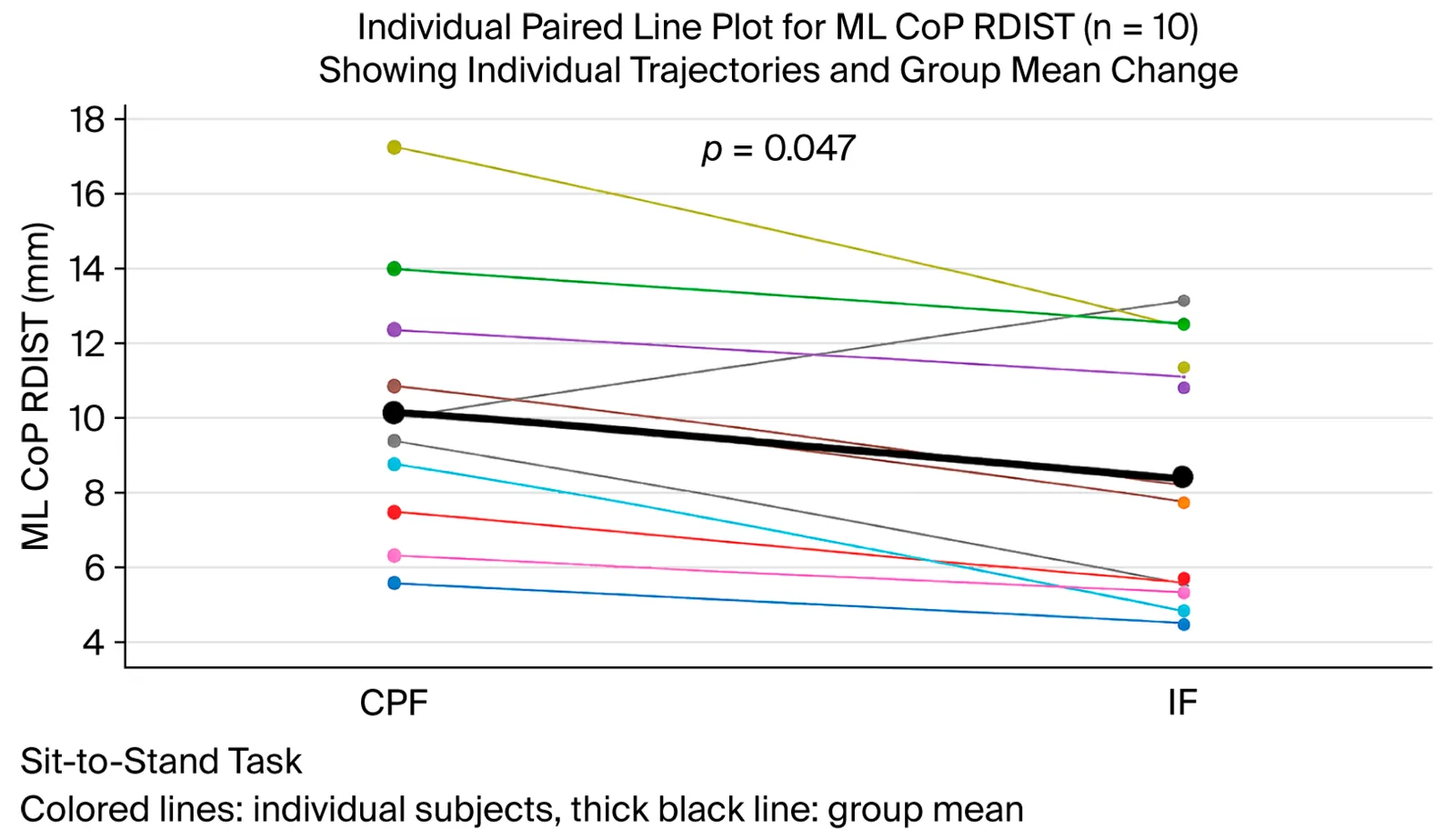
Individual paired responses in mediolateral root mean square distance (ML CoP RDIST) during the sit-to-stand task under the conventional prosthetic foot (CPF) and Intersection Foot^®^ (IF) conditions. Colored lines represent individual participants, and the thick black line represents the group mean.

The biomechanical variables captured during the sit-to-stand task are summarized in Table 2. During the sit-to-stand task, the IF condition showed a significant reduction in the ML RDIST of the CoP compared with the CPF condition (8.29 ± 3.32 mm vs. 10.20 ± 3.59 mm, *p* = 0.047, Hedges’ *g* = 0.68, 95% CI: 0.02 to 1.31). The AP range of CoM movement also significantly decreased in the IF condition (326.45 ± 47.40 mm vs. 345.51 ± 31.72 mm, *p* = 0.008, Hedges’ *g* = 0.77, 95% CI: 0.05 to 1.44). The ML MDIST of the CoP was lower in the IF condition than in the CPF condition, although the difference did not reach statistical significance (6.87 ± 2.93 mm vs. 8.45 ± 2.79 mm, *p* = 0.059, Hedges’ *g* = 0.57, 95% CI: −0.06 to 1.18). Individual paired data for ML CoP RDIST are presented in Figure 5, illustrating the within-subject responses between the CPF and IF conditions.

### 3.2. Standing with Eyes Open and Closed

The biomechanical outcomes during the eyes-open and eyes-closed standing tasks are summarized in Table 3. During the eyes-open standing task, the ML MDIST of the CoP was significantly higher in the IF condition compared with the CPF condition (0.39 ± 0.16 mm vs. 0.31 ± 0.14 mm, *p* = 0.038, Hedges’ *g* = −0.66, 95% CI: −1.29 to −0.01). This finding indicates greater mediolateral CoP sway in the IF condition. Additionally, the ML RDIST of the CoP demonstrated an increasing trend with a moderate effect size (0.44 ± 0.18 mm vs. 0.36 ± 0.16 mm, *p* = 0.059, Hedges’ *g* = −0.65, 95% CI: −1.27 to 0.004). No significant differences in CoM displacement were observed between the two conditions during eyes-open standing. In the eyes-closed condition, no statistically significant differences in CoP or CoM parameters were observed between the two prosthetic conditions.

### 3.3. Tandem Stance and Feet-Together Tasks

**Figure 6:**
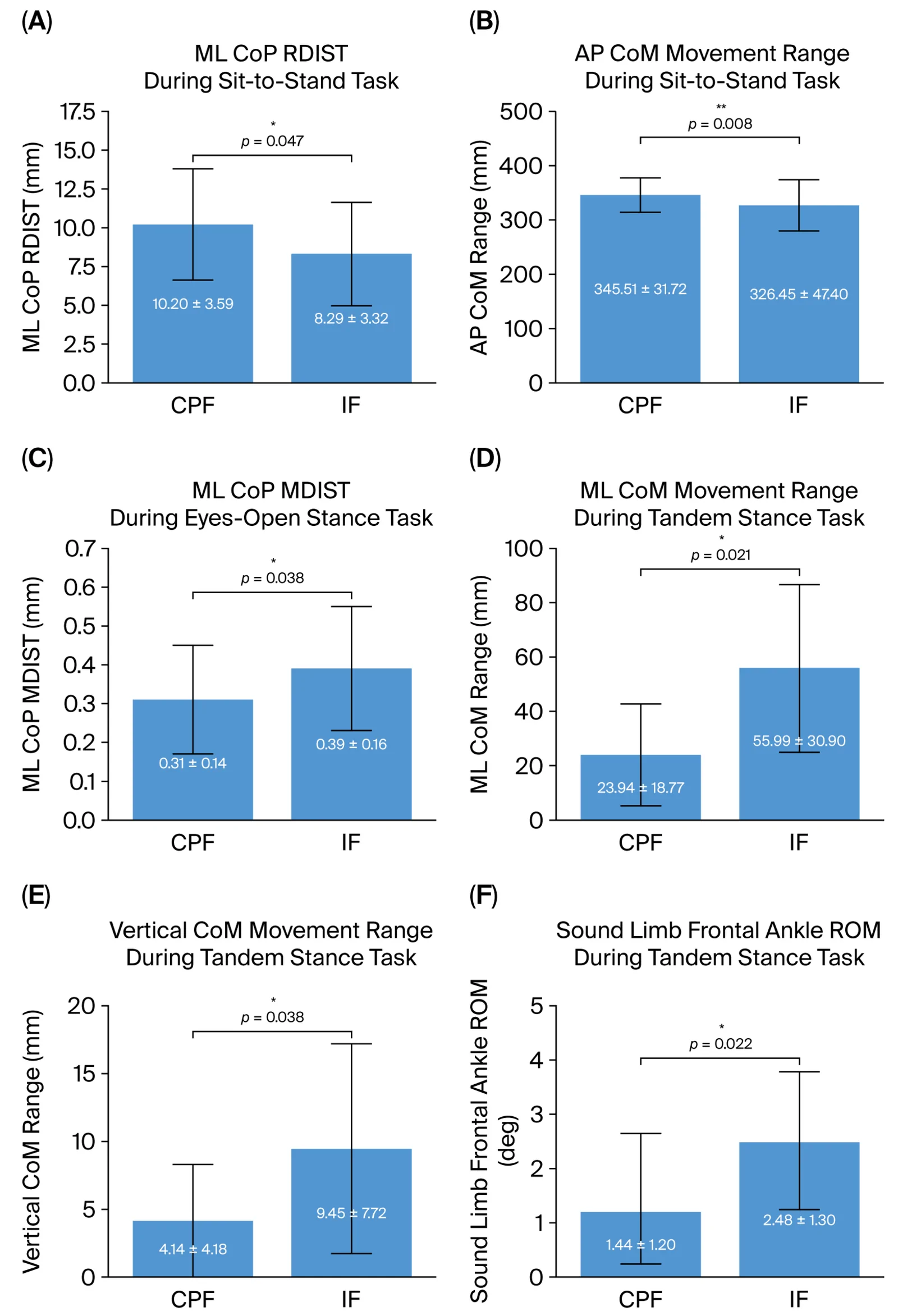
Summary of the principal biomechanical differences between the conventional prosthetic foot (CPF) and Intersection Foot^®^ (IF) conditions. Significant outcomes are shown for (**A**) mediolateral CoP RDIST during sit-to-stand, (**B**) anteroposterior CoM movement range during sit-to-stand, (**C**) mediolateral CoP MDIST during eyes-open standing, (**D**) mediolateral CoM movement range during tandem stance, (**E**) vertical CoM movement range during tandem stance, and (**F**) sound-side frontal-plane ankle ROM during tandem stance. Values are presented as mean ± standard deviation. * *p* < 0.05; ** *p* < 0.01.

The biomechanical variables captured during the tandem stance and feet-together standing tasks are presented in Table 4. During the tandem stance task, the IF condition exhibited significantly greater ML and vertical CoM ranges than the CPF condition. The ML range of CoM increased from 23.94 ± 18.77 mm to 55.99 ± 30.90 mm (*p* = 0.021, Hedges’ *g* = −0.78, 95% CI: −1.47 to −0.07), and the vertical range of CoM increased from 4.14 ± 4.18 mm to 9.45 ± 7.72 mm (*p* = 0.038, Hedges’ *g* = −0.73, 95% CI: −1.40 to −0.03). The ML ranges and ML RDIST of CoP also showed increasing trends with moderate effect sizes, although these differences were not statistically significant. Balance maintenance time tended to increase in the IF condition compared with the CPF condition (7.27 ± 7.00 s vs. 3.57 ± 3.13 s, *p* = 0.093, Hedges’ *g* = −0.50, 95% CI: −1.10 to 0.12). In addition, the frontal-plane ankle ROM on the sound side significantly increased in the IF condition (2.48 ± 1.30° vs. 1.44 ± 1.20°, *p* = 0.022, Hedges’ *g* = −0.82, 95% CI: −1.48 to −0.13). No significant differences were observed during the feet-together standing tasks. The principal significant biomechanical differences between the CPF and IF conditions across the sit-to-stand, eyes-open standing, and tandem stance tasks are summarized in Figure 6.

## 4. Discussion

This study examined the effects of the IF on balance control in individuals with transtibial amputation during selected BBS-derived tasks. The findings demonstrated task-dependent biomechanical adaptations rather than uniform improvements in postural stability. During sit-to-stand, the IF condition was associated with reduced ML CoP dispersion and AP CoM excursion. During eyes-open standing, ML CoP displacement increased without a significant change in CoM excursion. During tandem stance, the IF condition showed greater ML and vertical CoM excursions, a nonsignificant tendency toward a longer balance maintenance time, and increased frontal-plane ankle ROM on the sound side. No significant differences were observed during eyes-closed or feet-together standing.

During sit-to-stand, individuals with transtibial amputation often show asymmetric weight distribution and greater loading on the intact limb, particularly during the rising phase [31,32]. In this study, the IF condition significantly reduced the ML CoP RDIST and AP CoM excursions compared to the CPF condition. These changes suggest more controlled weight transfer during the transition from sitting to standing. Given that long-term prosthesis use may be associated with secondary physical conditions in the intact limb, reduced ML CoP dispersion during sit-to-stand may reflect a more constrained mediolateral postural response during dynamic weight transfer [33]. Because sit-to-stand represents a transitional task requiring dynamic postural control, the clearer changes observed during this task than during quiet standing suggest that the biomechanical effects of the IF may depend on the postural demands of the task.

The static standing tasks resulted in smaller changes. During eyes-open standing, the IF condition increased ML CoP displacement, whereas CoM excursions did not change significantly. Although greater CoP excursion during quiet standing may indicate increased postural sway [34], CoP displacement alone may not fully characterize postural balance control [35]. In the present study, the absence of a corresponding increase in CoM excursion suggests that the observed CoP response was not accompanied by greater whole-body movement. During eyes-closed standing, no significant differences in CoP or CoM parameters were observed between the two prosthetic conditions.

Tandem stance imposes a narrow base of support and is highly challenging for individuals with transtibial amputation because active ankle control is absent on the prosthetic side [36]. In this study, the IF condition was associated with significantly greater ML and vertical CoM excursions during the tandem stance. Although greater CoM excursion may represent increased whole-body sway, balance maintenance time showed only a nonsignificant tendency to increase and therefore should not be interpreted as evidence of improved stability. The increase in frontal-plane ankle ROM on the sound side further suggests greater involvement of sound-limb compensatory motion during this task. Such compensatory reliance may increase mechanical demands on the sound limb [32], which may contribute to secondary joint degeneration, including osteoarthritis [33,37]. In contrast, neither sagittal- nor frontal-plane prosthetic-side ankle ROM differed significantly between the CPF and IF conditions. Notably, nine of the ten habitual prosthetic feet were ESAR designs, and the IF is also an ESAR foot. Because ESAR feet primarily accommodate sagittal-plane loading through elastic deformation and energy storage and return [16,17], the shared sagittal-plane mechanical characteristics of the habitual ESAR feet and the IF may partly explain the absence of a significant difference in prosthetic-side sagittal-plane ROM. In addition, motion measured on the prosthetic side primarily reflects passive deformation of the prosthetic components rather than active neuromuscular joint motion. Therefore, any additional deformation associated with the specific structural configuration of the IF during static tandem stance may have been too small to produce a significant change in the measured ROM. No significant differences were observed during feet-together standing. Overall, these findings indicate that the biomechanical responses associated with the IF were task-dependent, with more pronounced changes during sit-to-stand and tandem stance than during quiet standing.

The habitual prosthetic feet used by the participants represented heterogeneous mechanical designs, including different ESAR configurations such as the Trias, Vari-Flex, and Pro-Flex. Previous studies have shown that differences among ESAR foot designs can influence gait and loading responses [37,38]. Therefore, the present comparison should not be interpreted simply as a comparison between a conventional non-ESAR foot and an ESAR foot. Rather, the IF differs from the habitual feet primarily in its specific asymmetric medial–lateral carbon-fiber configuration and X-shaped plantar structure, which are intended to permit mediolateral and torsional deformation under load. However, because the mechanical deformation, stiffness, and torsional characteristics of the individual habitual feet and the IF were not directly quantified, the contribution of these specific structural differences to the observed biomechanical responses cannot be determined.

In addition to prosthetic foot design, residual-limb morphology may influence the mechanical interaction between the residual limb and socket. Baharuddin et al. reported in a patient-specific finite-element study that tibial length and distal tibial bevel angle affected soft-tissue stress distribution at the residual limb–socket interface [39]. In the present cohort, residual tibial length varied across participants (9.1–20.1 cm; mean, 15.15 cm), whereas distal tibial bevel angle was not measured. Although these residual-limb characteristics were unchanged between the CPF and IF conditions within each participant, variability in residual tibial length and other unmeasured morphological features may have contributed to interindividual differences in the magnitude of the observed biomechanical responses.

From a clinical perspective, the present biomechanical changes should be interpreted cautiously. Establishing universal threshold values for CoP, CoM, and ankle ROM is challenging in individuals with lower-limb amputation because postural control measures may vary according to individual characteristics, prosthetic configuration, compensatory strategies, task demands, and measurement protocols. Previous studies in individuals with transtibial amputation have also used different CoP-, CoM-, and posturography-based measures across standing and balance tasks, further illustrating the task-dependent nature of balance assessment [2]. Therefore, the present findings should be interpreted as relative biomechanical differences between prosthetic conditions rather than against an established clinical cutoff. Although the observed task-dependent responses may provide insight into how prosthetic-foot characteristics influence postural control, these laboratory findings alone cannot establish improved real-world balance or reduced fall risk.

### Limitations

This pilot study has some limitations. First, the sample size was small (*n* = 10), reflecting the exploratory nature of the study and the practical difficulty of recruiting eligible individuals with unilateral transtibial amputation. In addition, all participants were male, and the cohort was predominantly older, with a relatively wide age range. Because age may influence postural control characteristics, age-related differences may have contributed to interindividual variability in the observed biomechanical responses. However, the small sample size precluded meaningful age-stratified or age-adjusted analyses. Future studies should therefore include larger and more diverse cohorts, including women and younger adults. The single-group pre–post design also involved a fixed testing order, with the habitual prosthetic foot assessed before the IF; therefore, potential order-, learning-, or time-related effects cannot be completely separated from the effects of the prosthetic foot condition.

Second, the baseline prosthetic conditions were heterogeneous. Nine of the ten participants were already using ESAR feet, and the habitual prosthetic feet differed in their mechanical designs. This represents an important limitation because the present study did not compare the IF with a single standardized control foot, thereby limiting attribution of the observed differences to specific features of the IF. Socket type was also not standardized across participants, and differences in residual-limb characteristics may have contributed to interindividual variability. Furthermore, the stiffness, torsional response, and mechanical deformation of the individual prosthetic feet were not directly quantified.

Third, the scope of the biomechanical assessment was limited. The force plate tasks were performed on a flat, rigid surface without externally imposed perturbations; therefore, the present findings cannot be generalized to reactive balance or postural responses to unexpected disturbances. In addition, clinical outcomes such as prospective falls and real-world balance performance were not assessed, preventing direct translation of the laboratory biomechanical findings into clinical benefit. Joint torque, joint power, and electromyographic activity were also not evaluated, limiting interpretation of the kinetic and neuromuscular mechanisms underlying the observed compensatory responses. Future studies should incorporate perturbation-based paradigms, including balance board assessments, as well as uneven or variable surfaces, and they should combine CoP and CoM measures with joint kinetics, muscle activity, standardized clinical balance assessments, and prospective fall-related outcomes.

## 5. Conclusions

In conclusion, the IF was associated with task-dependent biomechanical responses in individuals with unilateral transtibial amputation rather than uniform improvements in postural stability. During sit-to-stand, the IF reduced mediolateral CoP dispersion and anteroposterior CoM excursion, whereas during eyes-open standing, mediolateral CoP displacement increased without a corresponding change in CoM excursion. During tandem stance, greater mediolateral and vertical CoM excursions and increased frontal-plane ankle ROM on the sound side were observed, while prosthetic-side ankle ROM did not differ significantly between conditions. No significant differences were observed during eyes-closed or feet-together standing. These preliminary findings suggest that the effects of the IF depend on the postural demands of the task and may involve both changes in whole-body balance behavior and compensatory responses of the sound limb. Further studies with larger and more diverse populations, standardized comparator feet, and clinical and perturbation-based balance outcomes are needed to determine the functional and clinical relevance of these biomechanical changes.

## Figures and Tables

**Table 1:** Demographic and prosthetic characteristics of participants (*n* = 10).

Participants	Height (cm)	Mass (kg)	Age (yrs.)	Sex	Feet	Socket	RTL (cm)	TSA (yrs.)	K-Level	Suspension	Side
1	179.3	83.1	46	M	C-walk ^a^	TSB	9.1	27	3	Suction	Rt
2	171.2	85.5	61	M	Vari-flex ^b^	TSB	10.1	11	3	Pin	Lt
3	172.7	74.6	74	M	1D35 ^a^	TSB	20.1	51	3	Pin	Lt
4	173.1	92.8	54	M	HeraFT ^c^	TSB	16.2	7	3	Suction	Lt
5	167.5	62.7	74	M	Trias ^a^	TSB	17.9	51	2	Suction	Lt
6	171.4	70.8	75	M	C-walk ^a^	TSB	13.8	51	3	Pin	Rt
7	158.8	56.4	75	M	C-walk ^a^	TSB	13.9	3	3	Pin	Rt
8	167.3	60.35	76	M	1H38 ^a^	PTB	19.6	53	3	Cuff	Lt
9	178.8	73.9	62	M	Pro-flex ^b^	TSB	14.1	55	3	Suction	Lt
10	166.9	61.3	62	M	1D35 ^a^	TSB	16.7	39	2	Suction	Rt
Mean	170.7	72.15	65.9				15.15	34.8			

M, male; TSB, total surface bearing; PTB, patellar tendon bearing; RTL, residual tibial length; TSA, time since amputation; Lt, left; Rt, right. ^a^ Otto Bock, Duderstadt, Germany; ^b^ Össur, Reykjavik, Iceland; ^c^ Hugo Dynamics, Suwon, Republic of Korea.

**Table 2:** Biomechanical variables during sit-to-stand task.

Variables		Sit-to-Stand			
		CPF	IF	*p*	*g* (95% CI)
Range of CoP	AP (mm)	7.51 ± 5.77	6.22 ± 4.35	0.445	0.31 (−0.28 to 0.88)
	ML (mm)	10.79 ± 6.68	9.65 ± 3.83	0.386	0.23 (−0.35 to 0.80)
MDIST of CoP	AP (mm)	8.83 ± 2.57	8.56 ± 1.75	0.575	0.08 (−0.49 to 0.64)
	ML (mm)	8.45 ± 2.79	6.87 ± 2.93	0.059	0.57 (−0.06 to 1.18)
	Resultant (mm)	13.72 ± 2.68	12.17 ± 2.52	0.139	0.46 (−0.15 to 1.06)
RDIST of CoP	AP (mm)	10.45 ± 3.10	10.13 ± 2.38	0.878	0.07 (−0.50 to 0.64)
	ML (mm)	10.20 ± 3.59	8.29 ± 3.32	0.047 *	0.68 (0.02 to 1.31)
	Resultant (mm)	15.10 ± 3.09	13.39 ± 2.98	0.241	0.45 (−0.17 to 1.04)
Range of CoM	Axis *X* (mm)	345.51 ± 31.72	326.45 ± 47.40	0.008 **	0.77 (0.05 to 1.44)
	Axis *Y* (mm)	28.21 ± 9.07	24.44 ± 9.58	0.374	0.21 (−0.40 to 0.80)
	Axis *Z* (mm)	302.89 ± 34.12	303.26 ± 33.05	0.260	0.43 (−0.21 to 1.04)
Amputee ankle ROM	Axis *X* (°)	4.88 ± 2.03	4.22 ± 1.71	0.285	0.31 (−0.28 to 0.88)
	Axis *Y* (°)	1.93 ± 1.01	2.23 ± 1.00	0.445	−0.23 (−0.80 to 0.36)
Sound ankle ROM	Axis *X* (°)	11.67 ± 4.03	12.24 ± 3.28	0.241	−0.20 (−0.77 to 0.38)
	Axis *Y* (°)	3.67 ± 0.79	3.87 ± 1.05	0.508	−0.16 (−0.73 to 0.41)

CPF, conventional prosthetic foot; IF, intersection foot; AP, anteroposterior; CoM, center of mass; CoP, center of pressure; MDIST, mean distance; ML, mediolateral; RDIST, root mean square distance; ROM, range of motion. For CoM variables, the *X*, *Y*, and *Z* axes represent the anteroposterior, mediolateral, and vertical directions, respectively. For ankle ROM variables, the *X* and *Y* axes represent sagittal- and frontal-plane motions, respectively. Values are expressed as mean ± standard deviation. * *p* < 0.05, ** *p* < 0.01 indicate statistical significance. Hedges’ *g* values are presented with corresponding 95% confidence intervals (CIs).

**Table 3:** Biomechanical variables during the eyes-open and eyes-closed standing tasks.

Variables		Eyes Open				Eyes Closed			
		CPF	IF	*p*	*g* (95% CI)	CPF	IF	*p*	*g* (95% CI)
Range of CoP	AP (mm)	2.20 ± 0.76	2.22 ± 0.94	0.959	−0.02 (−0.59, 0.55)	4.01 ± 1.70	3.54 ± 1.43	0.799	0.17 (−0.40, 0.74)
	ML (mm)	1.17 ± 0.45	1.41 ± 0.54	0.169	−0.53 (−1.13, 0.10)	1.99 ± 0.65	2.17 ± 0.67	0.333	−0.27 (−0.84, 0.32)
MDIST of CoP	AP (mm)	0.54 ± 0.23	0.62 ± 0.36	0.374	−0.18 (−0.74, 0.40)	1.01 ± 0.45	0.91 ± 0.49	0.575	0.11 (−0.46, 0.68)
	ML (mm)	0.31 ± 0.14	0.39 ± 0.16	0.038 *	−0.66 (−1.29, −0.01)	0.49 ± 0.14	0.58 ± 0.18	0.139	−0.45 (−1.04, 0.16)
	Resultant (mm)	0.67 ± 0.27	0.80 ± 0.33	0.214	−0.36 (−0.94, 0.24)	1.22 ± 0.46	1.18 ± 0.48	0.646	0.05 (−0.52, 0.61)
RDIST of CoP	AP (mm)	0.64 ± 0.25	0.70 ± 0.38	0.575	−0.13 (−0.69, 0.45)	1.21 ± 0.54	1.05 ± 0.50	0.508	0.16 (−0.41, 0.73)
	ML (mm)	0.36 ± 0.16	0.44 ± 0.18	0.059	−0.65 (−1.27, 0.004)	0.59 ± 0.18	0.68 ± 0.21	0.169	−0.42 (−1.01, 0.18)
	Resultant (mm)	0.74 ± 0.28	0.87 ± 0.35	0.285	−0.30 (−0.88, 0.29)	1.37 ± 0.54	1.29 ± 0.49	0.575	0.08 (−0.49, 0.65)
Range of CoM	Axis *X* (mm)	4.41 ± 1.09	4.19 ± 1.37	0.374	0.24 (−0.37, 0.83)	3.02 ± 1.56	3.44 ± 2.34	0.859	−0.14 (−0.73, 0.45)
	Axis *Y* (mm)	1.84 ± 0.77	1.74 ± 0.62	0.594	0.17 (−0.43, 0.76)	0.95 ± 0.33	1.27 ± 0.67	0.139	−0.53 (−1.16, 0.12)
	Axis *Z* (mm)	0.46 ± 0.17	0.41 ± 0.12	0.173	0.39 (−0.24, 1.00)	0.45 ± 0.23	0.41 ± 0.13	0.314	0.27 (−0.35, 0.86)

CPF, conventional prosthetic foot; IF, intersection foot; AP, anteroposterior; CoM, center of mass; CoP, center of pressure; MDIST, mean distance; ML, mediolateral; RDIST, root mean square distance. For the CoM and kinematic variables, the *X*, *Y*, and *Z* axes represent the anteroposterior, mediolateral, and vertical directions, respectively. Values are expressed as mean ± standard deviation. * *p* < 0.05. Hedges’ *g* values are presented with corresponding 95% confidence intervals (CIs).

**Table 4:** Biomechanical variables during tandem stance and feet-together tasks.

Variables		Tandem Stance				Feet Together			
		CPF	IF	*p*	*g* (95% CI)	CPF	IF	*p*	*g* (95% CI)
Range of CoP	AP (mm)	63.54 ± 46.61	107.14 ± 66.35	0.114	−0.52 (−1.12, 0.11)	3.10 ± 0.82	2.60 ± 0.98	0.594	0.38 (−0.24, 0.99)
	ML (mm)	19.18 ± 10.42	27.39 ± 10.69	0.074	−0.71 (−1.35, −0.04)	2.65 ± 1.62	2.32 ± 1.19	0.515	0.21 (−0.39, 0.80)
MDIST of CoP	AP (mm)	12.27 ± 8.98	12.93 ± 6.31	0.646	−0.07 (−0.63, 0.50)	0.82 ± 0.29	0.68 ± 0.31	0.594	0.24 (−0.37, 0.84)
	ML (mm)	4.54 ± 2.03	5.66 ± 1.88	0.169	−0.43 (−1.02, 0.18)	0.67 ± 0.42	0.64 ± 0.36	0.767	0.11 (−0.49, 0.70)
	Resultant (mm)	14.02 ± 9.12	15.13 ± 6.13	0.445	−0.12 (−0.68, 0.46)	1.20 ± 0.36	1.03 ± 0.46	0.214	0.37 (−0.26, 0.97)
RDIST of CoP	AP (mm)	14.83 ± 10.47	18.50 ± 9.34	0.285	−0.27 (−0.84, 0.32)	0.95 ± 0.31	0.79 ± 0.34	0.678	0.28 (−0.33, 0.88)
	ML (mm)	5.34 ± 2.47	6.80 ± 2.15	0.074	−0.50 (−1.10, 0.12)	0.78 ± 0.48	0.74 ± 0.40	0.767	0.11 (−0.48, 0.70)
	Resultant (mm)	16.16 ± 10.39	20.05 ± 9.08	0.285	−0.29 (−0.87, 0.30)	1.33 ± 0.39	1.12 ± 0.48	0.214	0.37 (−0.25, 0.98)
Range of CoM	Axis *X* (mm)	29.20 ± 43.68	53.45 ± 51.51	0.086	−0.49 (−1.12, 0.16)	3.58 ± 1.99	2.93 ± 1.02	0.374	0.34 (−0.28, 0.94)
	Axis *Y* (mm)	23.94 ± 18.77	55.99 ± 30.90	0.021 *	−0.78 (−1.47, −0.07)	3.61 ± 2.68	3.36 ± 2.18	0.859	0.11 (−0.49, 0.70)
	Axis *Z* (mm)	4.14 ± 4.18	9.45 ± 7.72	0.038 *	−0.73 (−1.40, −0.03)	0.48 ± 0.16	0.41 ± 0.15	0.214	0.41 (−0.23, 1.02)
Amputee ankle ROM	Axis *X* (°)	0.47 ± 0.26	0.66 ± 0.53	0.646	−0.26 (−0.83, 0.32)	-	-	-	-
	Axis *Y* (°)	0.27 ± 0.13	0.43 ± 0.34	0.169	−0.41 (−1.00, 0.19)	-	-	-	-
Sound ankle ROM	Axis *X* (°)	1.54 ± 1.43	1.70 ± 0.91	0.508	−0.08 (−0.64, 0.49)	-	-	-	-
	Axis *Y* (°)	1.44 ± 1.20	2.48 ± 1.30	0.022 *	−0.82 (−1.48, −0.13)	-	-	-	-
Maintenance time (s)		3.57 ± 3.13	7.27 ± 7.00	0.093	−0.50 (−1.10, 0.12)	-	-	-	-

CPF, conventional prosthetic foot; IF, intersection foot; AP, anteroposterior; CoM, center of mass; CoP, center of pressure; MDIST, mean distance; ML, mediolateral; RDIST, root mean square distance; ROM, range of motion. For CoM variables, the *X*, *Y*, and *Z* axes represent the anteroposterior, mediolateral, and vertical directions, respectively. For ankle ROM variables, the *X* and *Y* axes represent sagittal- and frontal-plane motions, respectively. Values are expressed as mean ± standard deviation. * *p* < 0.05. Hedges’ *g* values are presented with corresponding 95% confidence intervals (CIs). Ankle ROM and balance maintenance time were analyzed for the tandem stance task only.

## Data Availability

Data will be made available on reasonable request from the corresponding author.

## Abbreviations

The following abbreviations are used in this manuscript:

ESAR	Energy-Storing-and-Return
ML	Mediolateral
BBS	Berg Balance Scale
CoP	Center of Pressure
CoM	Center of Mass
AP	Anteroposterior
MDIST	Mean Distance
RDIST	Root Mean Square Distance
ROM	Range of Motion
TSB	Total Surface Bearing
PTB	Patellar Tendon Bearing
EMG	Electromyography
